# Biogeochemical, Isotopic and Bacterial Distributions Trace Oceanic Abyssal Circulation

**DOI:** 10.1371/journal.pone.0145299

**Published:** 2016-01-13

**Authors:** Angelo Rubino, Manuel Bensi, Dagmar Hainbucher, Davide Zanchettin, Francesca Mapelli, Nives Ogrinc, Davide Marchetto, Sara Borin, Vanessa Cardin, Vesna Fajon, Milena Horvat, Carla Taricco, Franco Baldi

**Affiliations:** 1 Dipartimento di Scienze Ambientali, Informatica e Statistica, Università Ca' Foscari di Venezia, Campus Scientifico, Via Torino 155, 30172 Mestre, VE, Italy; 2 Istituto Nazionale di Oceanografia e di Geofisica Sperimentale, OGS, B.go Grotta Gigante 42/c, Sgonico, Trieste, Italy; 3 CEN, Institut für Meereskunde, University of Hamburg, Bundesstraße 53, 20146, Hamburg, Germany; 4 Dipartimento di Scienze per gli Alimenti, la Nutrizione e l'Ambiente, Università degli Studi di Milano, Milan, Italy; 5 Department of Environmental Sciences, “Jožef Stefan” Institut, Jamova 39, 1000, Ljubljana, Slovenia; 6 Dipartimento di Scienze Molecolari e Nanosistemi, Università Ca' Foscari di Venezia, Campus Scientifico, Via Torino 155, 30172, Mestre, VE, Italy; 7 Dipartimento di Fisica, Università di Torino, Torino, Italy; 8 Osservatorio Astrofisico di Torino, INAF, Pino Torinese, Italy; University of Aveiro, PORTUGAL

## Abstract

We explore the possibility of tracing routes of dense waters toward and within the ocean abyss by the use of an extended set of observed physical and biochemical parameters. To this purpose, we employ mercury, isotopic oxygen, biopolymeric carbon and its constituents, together with indicators of microbial activity and bacterial diversity found in bottom waters of the Eastern Mediterranean. In this basin, which has been considered as a miniature global ocean, two competing sources of bottom water (one in the Adriatic and one in the Aegean seas) contribute to the ventilation of the local abyss. However, due to a recent substantial reduction of the differences in the physical characteristics of these two water masses it has become increasingly complex a water classification using the traditional approach with temperature, salinity and dissolved oxygen alone. Here, we show that an extended set of observed physical and biochemical parameters allows recognizing the existence of two different abyssal routes from the Adriatic source and one abyssal route from the Aegean source despite temperature and salinity of such two competing sources of abyssal water being virtually indistinguishable. Moreover, as the near-bottom development of exogenous bacterial communities transported by convectively-generated water masses in the abyss can provide a persistent trace of episodic events, intermittent flows like those generating abyssal waters in the Eastern Mediterranean basin may become detectable beyond the availability of concomitant measurements.

## Introduction

Oceanic deep penetrating convection strongly influences global climate as it transforms the characteristics of large volumes of water [1]. It occurs episodically, and the dense waters it produces are spread throughout the oceans by near-bottom overflows, which can be episodic as well [2]. Routes and variability of these currents shape the properties of the ocean abyss, including its heat content distribution and variability [1,3]. However, a clear identification of abyssal water origin and circulation may be unviable when this water originates from competing sources possessing similar characteristics and/or it is found only episodically in sampled deep sites [4,5].

A large shift in the oceanic circulation of the Eastern Mediterranean Sea took place in the late 1980s [6]. As a result of a still unclear chain of meteorological, hydrologic and oceanographic events, deep waters of Aegean origin replaced colder and fresher waters of Adriatic origin in the Ionian abyss. Since then, a large variability characterizes the deep water structure and circulation of both the Eastern and the Western Mediterranean [7,8]. In particular, deep waters of Adriatic origin got saltier and warmer in recent years [4,5]. As a consequence, the difference in the characteristics of the two competing sources of deep Ionian waters has become vanishingly small and the possibility of tracing their different patterns and variability using classical methods rather subtle.

This urges the explorative assessment of alternative experimental methodologies in the field of physical oceanography of the abyss. Here, we use bottom biogeochemical, isotopic and bacterial distributions in an attempt to trace oceanic abyssal circulation patterns in the Eastern Mediterranean Sea.

## Materials and Methods

**Fig 1:**
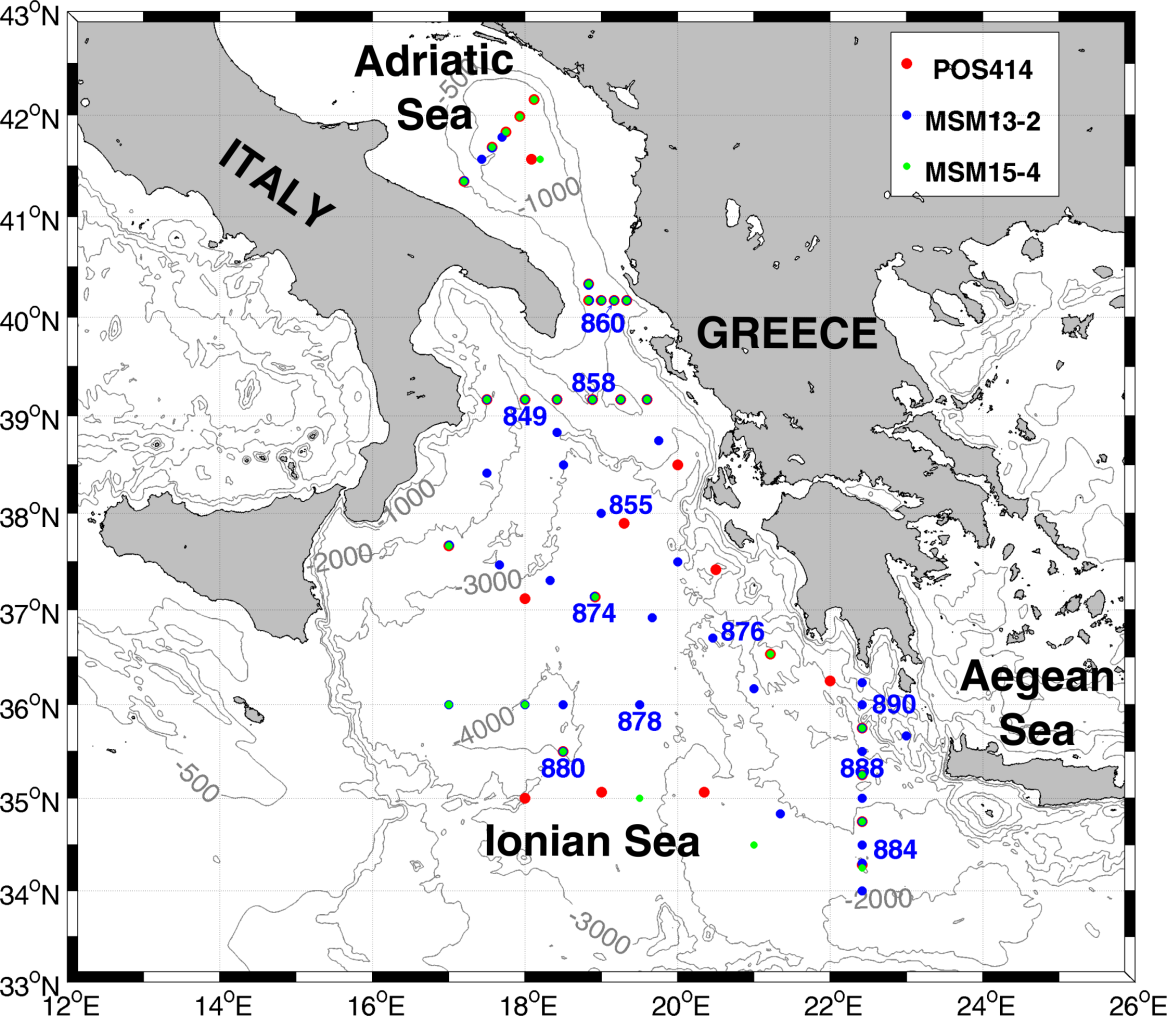
Bathymetry encompassing the Euro-African Mediterranean Sea where the data used in our investigation were collected. Samples from the different cruises are marked by different dot colours (red: POS414; blue MSM 13–2; green: MSM 15–4). Only the positions of the stations actually considered are indicated in the map.

Our attempt is based on measurements carried out in the abyssal and bottom layers of Adriatic and Ionian seas, in the Euro-African Mediterranean Sea, during October 2009, July 2010 (Research Vessel Maria Sybilla Merian, cruises MSM13-2 and MSM15-4, see [5]), and June 2011 (Research Vessel Poseidon, cruise POS 414, see [9]) (Fig 1). Temperature and conductivity data were obtained by means of a Conductivity-Temperature-Depth (CTD) rosette with the bottom-deep casts within ~10–12m of the seabed. Potential Temperature (θ), Salinity (S), and Potential Density Anomaly (σ_θ_) were calculated from each original in-situ data set. Dissolved Oxygen (DO) was measured using a Seabird sensor SBE 43 mounted on the CTD rosette and it was determined in parallel using the Winkler method [10]. Data were calibrated and averaged every 1 dbar with overall accuracies within 0.002°C for temperature, 0.005 for salinity and 2% of saturation for DO.

Isotopic oxygen (δ^18^Ο) measurements were performed following the CO_2_-H_2_O equilibration procedure described in [11]. The isotopic composition of oxygen was reported using the “δ notation" in parts-per-thousand deviation relative to a standard:

$$\delta^{18}O=\left(\frac{R_{sample}-R_{STD}}{R_{STD}}\right)\;x\;1000$$

where R_sample_ and R_STD_ are ^18^O/^16^O isotope ratios in sample and international standard, respectively. VSMOW (Vienna Standard Mean Ocean Water) was used as internal standard. Typical uncertainties at 1 standard deviation (σ) are ±0.1‰.

Total mercury (THg) was determined after immediate acidification (1% suprapur concentrated HCl). Firstly, Hg species in samples were oxidized by BrCl and by exposure to UV (Ultra violet) light. Excess bromine and chlorine were removed by addition of NH_2_OH^.^HCl. Oxidized Hg was then reduced by SnCl_2_, and Hg^0^ amalgamated on a gold trap followed by thermal desorption and detection in an LDC Milton Roy CV AAS mercury analyzer. The limit of detection was 0.5 pM (i.e., pico molar) calculated on the basis of 3σ of the reagent blank [12].

Biochemical composition of particulate organic matter was determined after seawater filtration performed through a pre-combusted glass-fiber filter. Carbohydrates (CHO) and proteins (PRT) concentrations were estimated after treatment of particulate organic matter on GF/F filters as demonstrated for Antarctic sediments [13]. Lipids (LIP) were determined by mean of fluorescence measure of Nile red, a fluorescent hydrophobic probe. Concentrations of CHO, PRT and LIP were then transformed in biopolymeric carbon (BPC) equivalent concentrations.

Extracellular β-glucosidase (GLU) and amimopeptidase (AMPT) activity was estimated in particulate organic matter (POM) through fluorescence determination of methylumbelliferone (MUF) and methylcoumarinylamide (MCA) produced during the breakdown of β-D-glucopyranoside-MUF and leucine-MCA substrata, respectively.

A volume of 4–5 L of bottom water was collected at 17 stations during the MSM 13–2 cruise for molecular microbiology analyses. DNA extraction was performed from filtered cells [14,15]. To describe the structure of bacterial communities Automated Ribosomal Intergenic Sequence Analysis (ARISA)-fingerprinting analysis was performed on a standard amount of DNA on each sample by applying established protocols using the primer set ITSF, 5’-GTC GTA ACA AGG TAG GCC GTA-3’ and ITSReub, 5’-GCC AAG GCA TCC ACC-3’ [16]. ARISA fingerprints were binned and statistically analyzed using the Microsoft Excel XLSTAT software (Addinsoft Inc., New York, NY, USA). Cluster analyses were performed using the StatSoft Statistica 6.0 software.

## Results and Discussion

**Fig 2:**
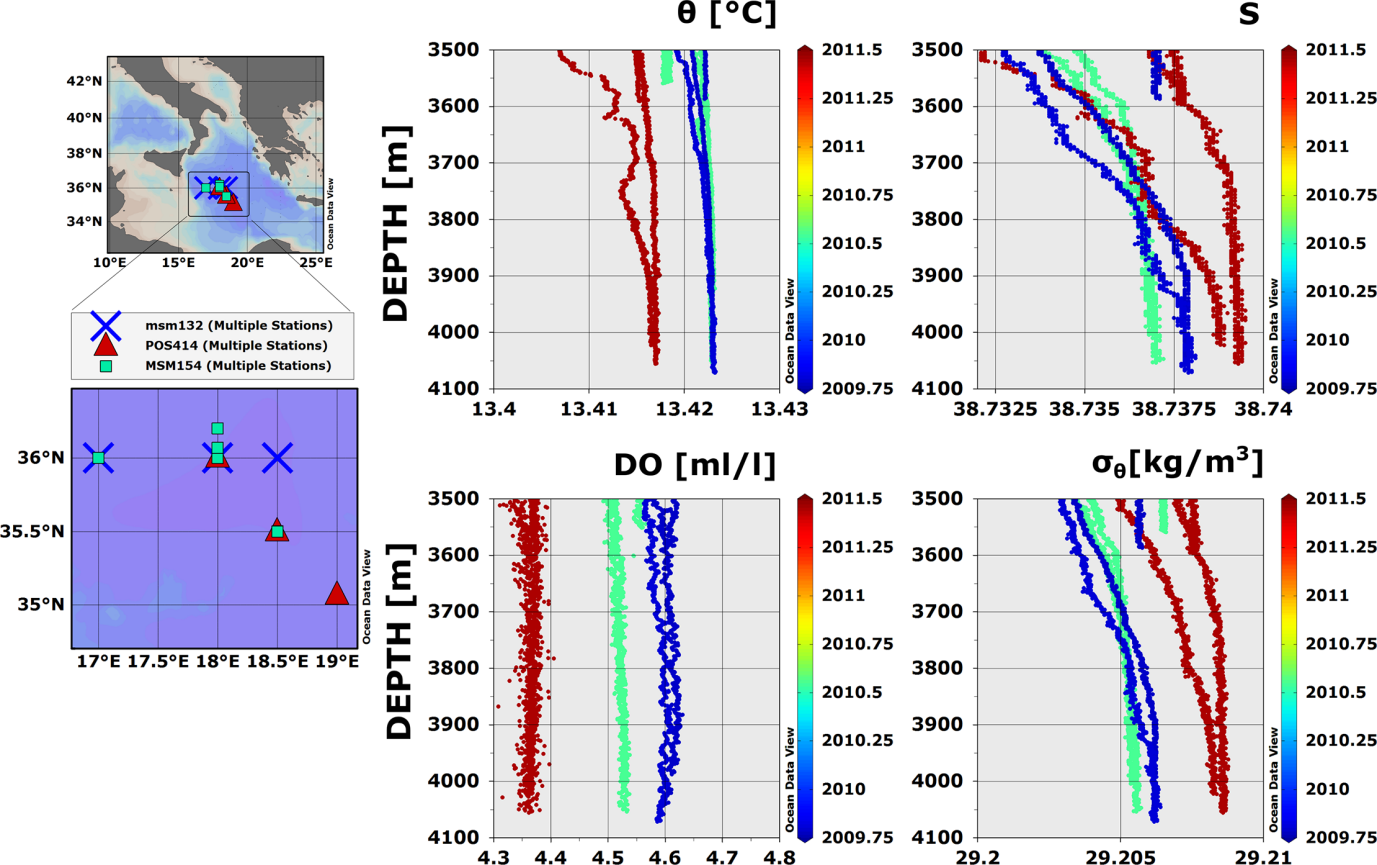
Vertical distribution of geophysical quantities in the abyssal Ionian Sea. Potential Temperature (θ,) Salinity (S), Dissolved Oxygen (DO), and Potential Density Anomaly (σ_θ_) were measured in the abyssal plain of the Ionian Sea in October 2009, July 2010 and June 2011. The data refers to depths > 3500 m. Colours individuate the different cruises (red: POS414; blue MSM 13–2; green: MSM 15–4). The vertical profiles refer to the locations mapped in the left panels (colour code is the same as for the right panels).

The vertical distributions of thermohaline properties and dissolved oxygen for selected stations of the Ionian abyssal plain (maximum depth circa 4100 m) measured during cruises MSM13-2, MSM15-4, and POS 414, are shown in Fig 2. The near-bottom values of θ and S, and hence σ_θ_, measured 500–600 m above the bottom underwent only minor variations through the three cruises. In the layer between 3500 m depth and the bottom, θ varied of about 0.01°C, and S of about 0.008. Instead, the near-bottom distribution of Dissolved Oxygen (DO) underwent a much larger variation (about 0.3 ml/l). The observed distributions reveal the presence of two distinct water masses in the Ionian abyssal layer: A more recent, denser bottom water coexisting with an older water [5]. In principle, a detailed knowledge of the history of the distribution of thermohaline parameters in the broader oceanic region encompassing the Ionian Basin could allow discerning the origin of the two water masses [5,7]. In practice, however, the measured local interannual variability can be vanishingly small at these large depths (Fig 2) [4,5,7]. Furthermore, differences in water properties at the sources of Adriatic and Aegean bottom waters—the two water masses mainly contributing to the Ionian abyssal distribution—have also become small in recent years due particularly to salinification and heating of Adriatic waters [17]. The latter phenomena have been attributed—among other things—to changes in larger-scale modes of oceanic variability [6,18].

**Fig 3:**
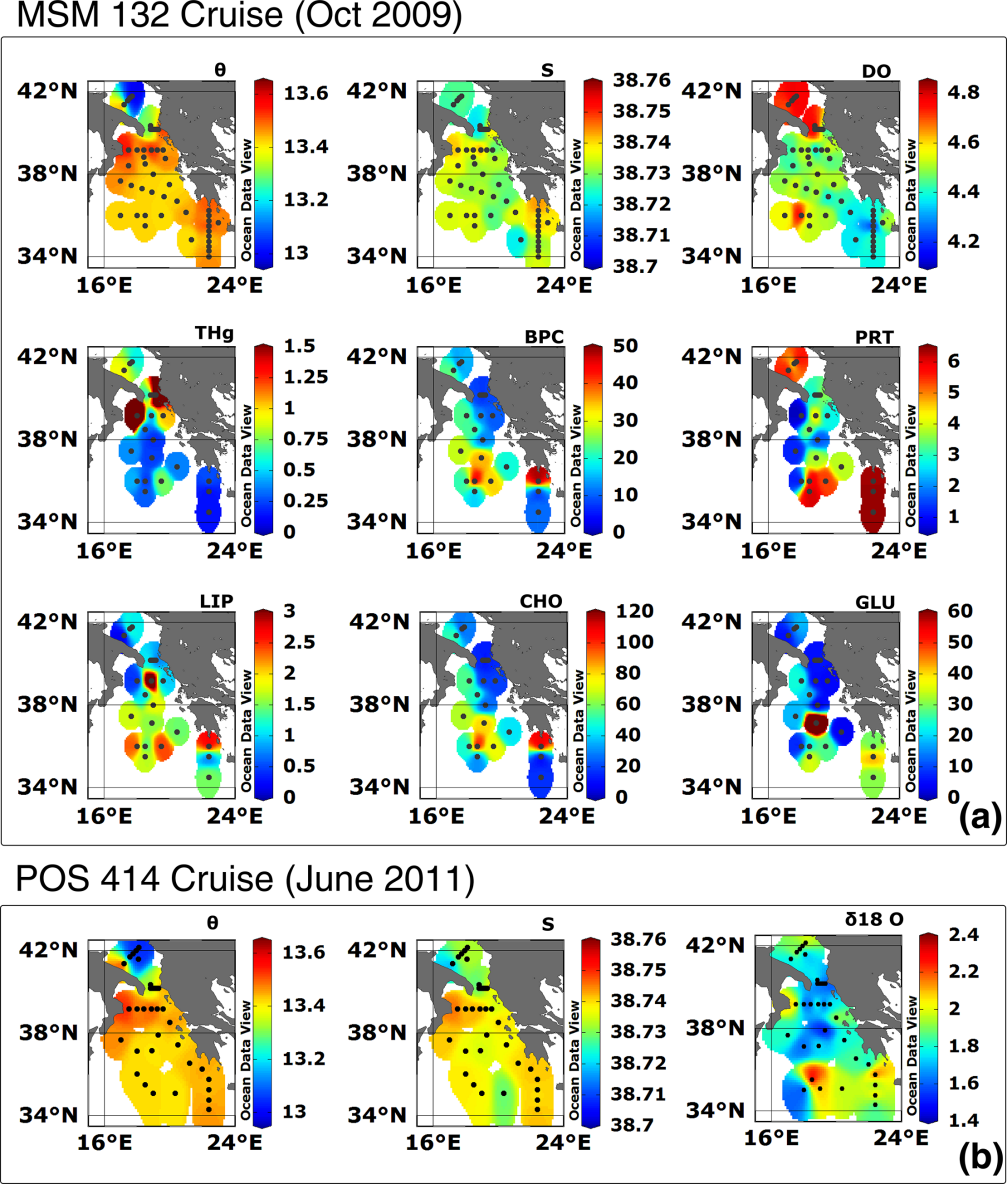
Bottom distribution of geophysical and biogeochemical parameters in the Eastern Mediterranean Sea. (**a**) Bottom distributions of θ, S, DO, THg, BPC and its constituents PRT, CHO and LIP, as well as enzymes GLU collected in 21 sites in the southern Adriatic basin and Ionian abyssal plain during MSM 13–2. (**b**) Bottom distributions of θ, S, and δ^18^Ο observed in the same region during POS 414. Data refer to the bottom-deep casts at depth within ~10–12 m above the seabed (see methods). Interpolation was performed with the weighted-average gridding algorithm of the software Ocean Data View.

**Fig 4:**
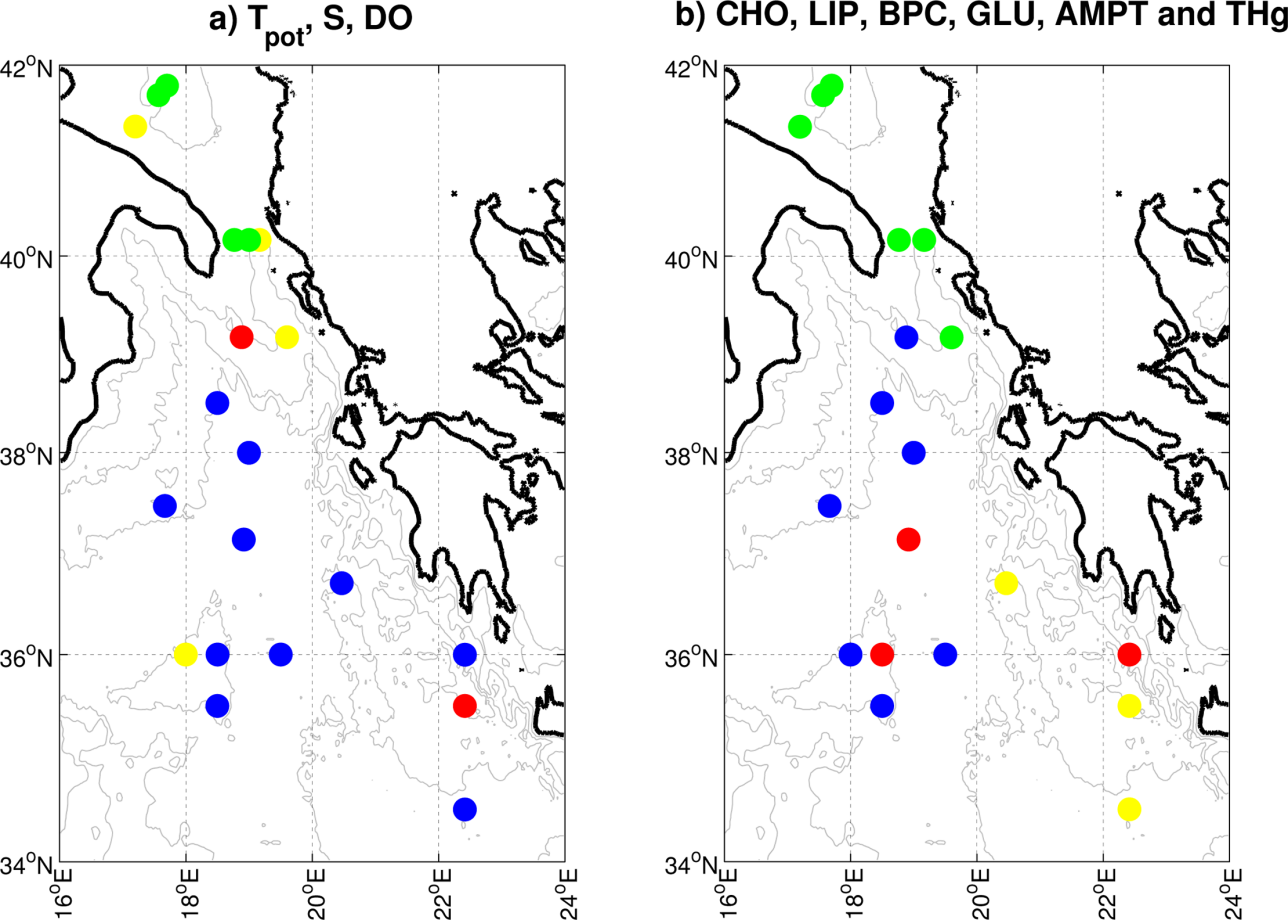
Grouping of the Eastern Mediterranean Sea bottom waters according to geophysical and biogeochemical parameters. Data are standardized Potential Temperature, S and DO data (**a**) and standardized CHO, LIP, BPC, GLU, AMPT and THg data (**b**). The grouping is based on a K-means clustering algorithm with four clusters. In each panel, the different colours identify the stations that belong to the same group.

The bottom distributions of θ, S, and DO in October 2009 (Fig 3A, upper panels, see S1 Dataset) identify the presence of deep waters of Adriatic origin, confined in the almost meridionally-oriented channel located along the Greek shelf being exported southward: Comparatively low values of both θ and S, together with large values of DO, identify this overflow. Larger values of θ and S and lower values of DO, instead, can be ascribed to older water masses, likely of Aegean origin, confined in the easternmost part of the Ionian Sea. However, beside the above mentioned regions, the distribution of θ, S, and DO does not allow for a straightforward water mass classification in the central Ionian abyss. This fact can be expressed more objectively using a cluster analysis. The grouping visible in Fig 4A shows a large variability, mostly localized in the approaches to the Strait of Otranto (there, three clusters can be found, see the green, yellow and red dots), which reflects different dynamical processes known to exist in the region. These include near-shore currents, open-ocean convection, and intermediate intrusions of Adriatic, Aegean, and Atlantic waters. The grouping further highlights variability near the Ionian approaches to the Cretan Sea, but it shows also a much more homogenous region in the central Ionian abyss (one cluster dominates here, see blue dots).

In the latter region, an attempt can be undertaken to obtain a more detailed information on the local abyssal water masses using observed biogeochemical (THg in POM, BPC with its constituent PRT, LIP and CHO, see S1 Dataset) and microbial diversity parameters. In the following, some of these parameter distributions will be discussed individually, as they seem to robustly reflect known characteristics of observed near-bottom distributions of physical oceanographic parameters. Other observed parameter distributions, instead, will be only interpreted within a multivariate cluster approach.

Large THg values are known to characterize both the western and the eastern flank of the Adriatic basin [19,20] due to riverine inputs [21] and mining in the Idrija district (Western Slovenia) [22]. THg is maximum along the approaches to the Strait of Otranto and in the north-western Ionian Sea, and it is above average along the eastern flank of the northernmost Ionian basin (Fig 3A). THg seems thus to follow the two known exporting routes of deep Adriatic waters toward the Ionian abyss [4,7]. A local maximum in the Ionian abyssal plain (St. 878 MSM 13–2) seemingly marks the onset of the deepest horizons where waters of Adriatic origin accumulate. The presence of two coastal local maxima (St. 860 and 849 MSM 13–2), where THg reaches values around 4.0 ng/l (Stations 849, 860) possibly reflects local anthropogenic contamination.

BPC is the most labile of total organic carbon and reacts with DO. Fig 3A suggests that BPC and DO are positively correlated in the Adriatic area, where water masses are mostly recent, while they are negatively correlated in the southern abyssal Ionian, where older water masses are present. Furthermore, the measured relative contributions of CHO, PRT and LIP to BPC, which amounts to 88.1%, 8.7% and 3.2%, respectively, suggest the polysaccharide origin of BPC due to nutrient depletion and environmental stress. The spatial distribution of CHO (Fig 3A) shows a mostly zonal pattern in the central and easternmost Ionian basin where larger values dominate, and an almost meridional path of relatively low values. LIP and BPC show instead a path possibly reflecting the route along which Adriatic abyssal water is typically exported toward the Ionian basin.

**Fig 5:**
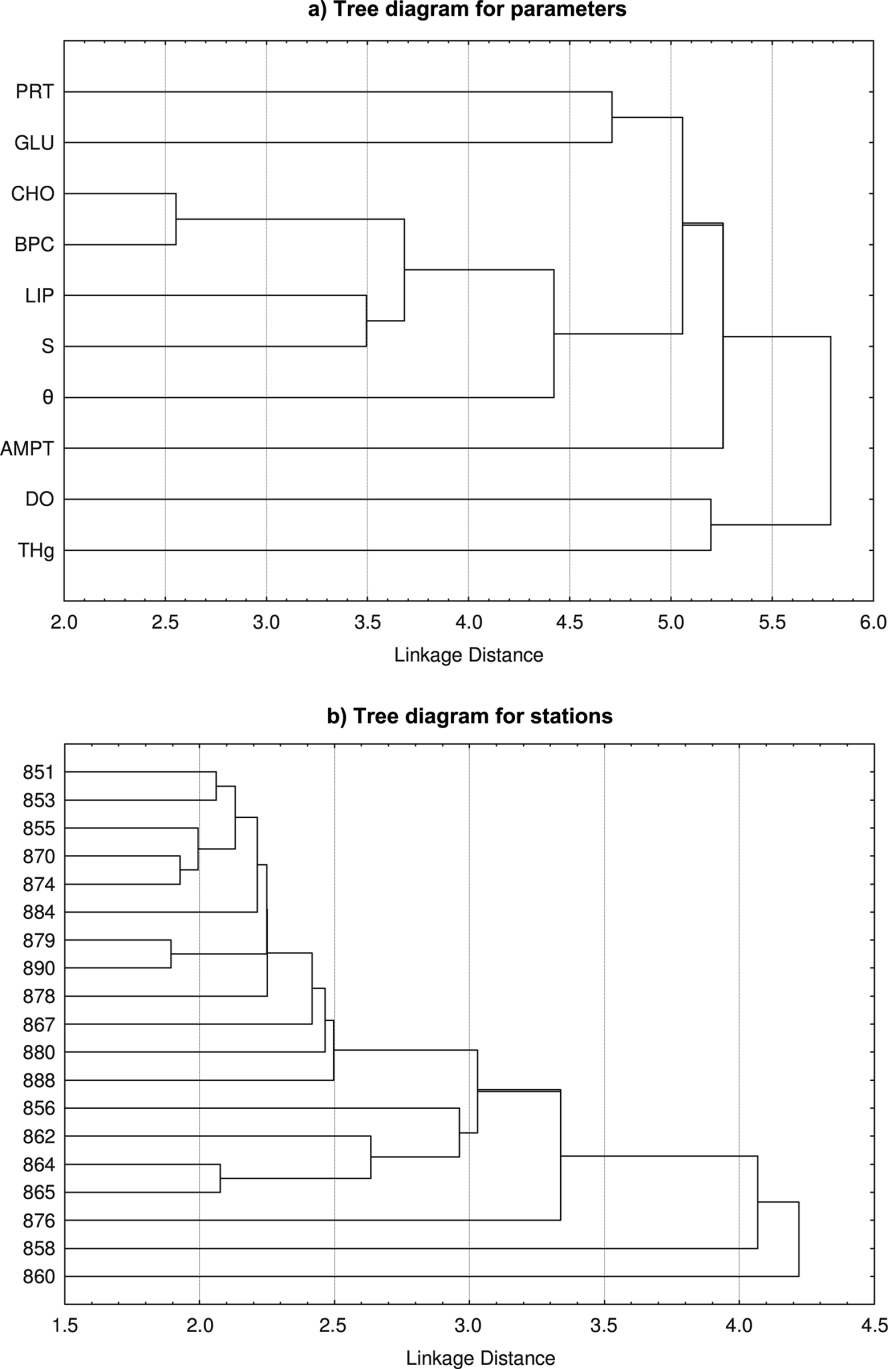
Grouping of parameters and stations in the Eastern Mediterranean abyss. The analysis is performed on parameters (a) and stations (b) of MSM 13–2 based on all available standardized physical and biogeochemical parameters. The grouping is based on a tree clustering algorithm with single linkage amalgamation rule.

Visual inspection is supported by a cluster analysis. In particular, Fig 5 shows tree clusters of Eastern Mediterranean near-bottom waters grouping all available parameters (panel a) and stations (panel b) included in the MSM 13–2 dataset (see S1 Dataset). We do not note a close one-by-one relation between individual parameters, except between CHO and BPC (r_CHO-BPC_ = 0.82, p<0.05) and, on a larger distance, between LIP and S (Fig 5A, r_LIP-S_ = 0.66, p<0.05). DO and THg share the smallest similarity with the remaining parameters and, although to a lesser extent, between themselves. Clustering of the sampling stations based on all available parameters similarly highlights the presence of stations with very distinct characteristics (stations 860, 858 and 876, possibly related to the high DO and THg values measured in these stations). Small distances are generally found between geographically close stations, reflecting the role of locally robust features. However, the statistical procedure does not clearly reveal the existence of a few basin-scale clusters. The shape of the tree diagram rather reflects the progressive transformation of water mass properties across the basin, whose spatial complexity remains then dominated by rather local characteristics.

A K-means clustering algorithm with the same model parameters used to generate Fig 4A demonstrates that the bulk biogeochemical data captures Adriatic waters protruding toward the Ionian abyss (green dots in Fig 4B) despite their great heterogeneity in physical properties (compare Fig 4A and 4B), and waters of Aegean origin also protruding toward the Ionian abyss (yellow and red dots in Fig 4B). The same clustering algorithm identifies water masses with different biogeochemical properties in the central Ionian abyss (Fig 4B), where physical parameters would lead to non-distinguishable water masses (Fig 4A). Based on the biogeochemical classification, the closest pair of groups includes the Adriatic cluster (green) described above and an almost meridional cluster (blue) in the central/western Ionian region (minimum Euclidean distance of 0.847). Dynamically, we tentatively interpret this result as an almost southward propagation and transformation (i.e., mixing with Aegean waters protruding northward in the Ionian Sea) of water masses of Adriatic origin. The data in the central and easternmost Ionian basin are separated into two groups (yellow and red) whose biogeochemical properties are comparatively more distant than those in the Adriatic and in the western Ionian basin (minimum Euclidean distance of 1.478). A possible interpretation of this fact is that the propagation of abyssal Aegean waters has been slower/weaker in recent years, leading to a larger biogeochemical variability in the region.

δ^18^Ο in the ocean is an excellent tracer for water masses because, far from the surface, it can be considered conservative and non-dynamical [23]. The bottom distributions of θ and S observed during the POS 414 cruise (Fig 3B) highlight, through their relatively low values, a trace of abyssal waters of Adriatic origin spreading on an almost meridional route consistent with the spreading observed in the 2009 and 2010 cruises (not shown). This feature of the thermohaline patterns is also apparent in the observed distribution of δ^18^Ο (Fig 3B), which shows a mostly zonal path of comparatively higher values intercepting the meridional route of lower values possibly associated with water of Adriatic origin.

**Fig 6:**
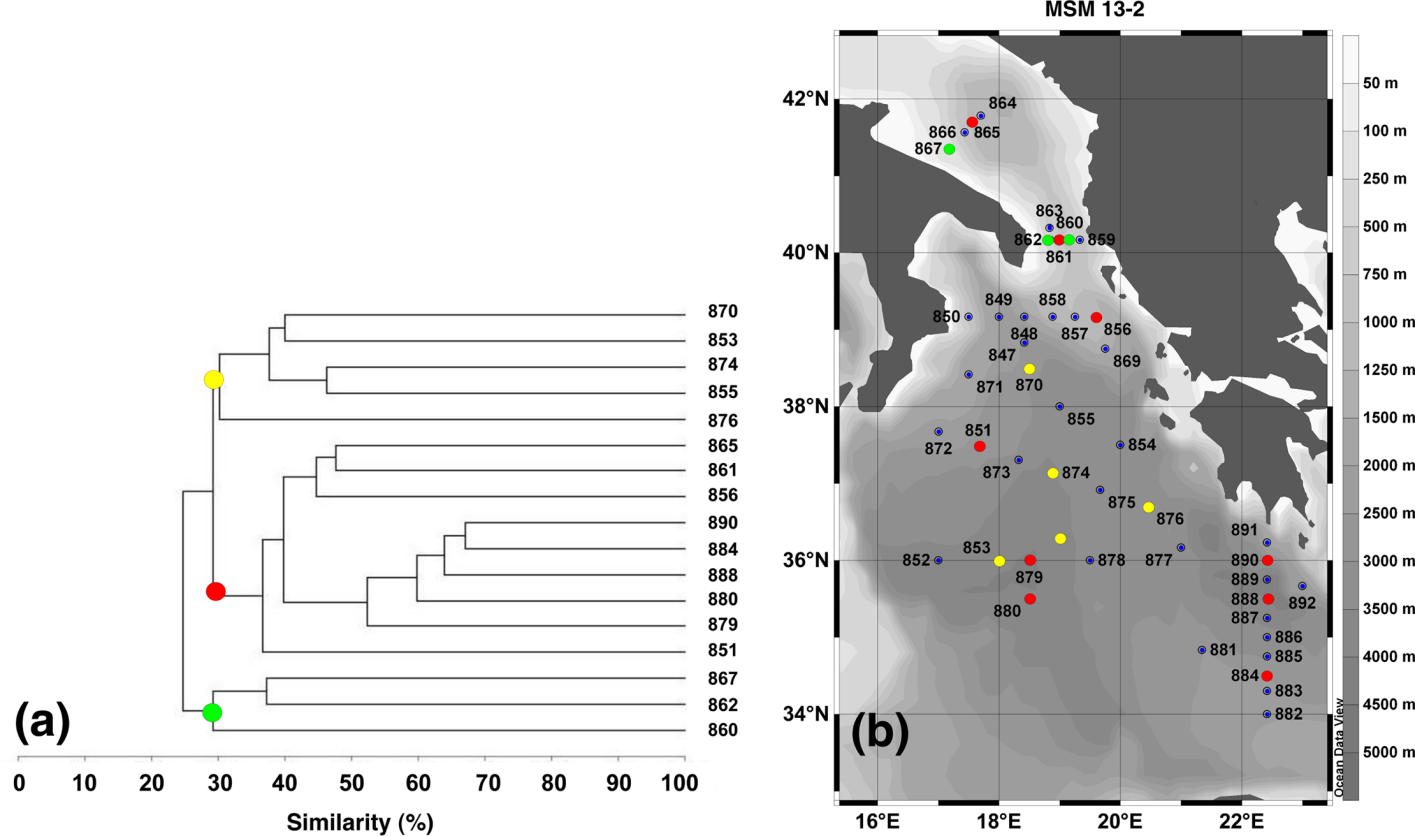
Grouping of the Eastern Mediterranean Sea bottom waters according to bacterial diversity. (a) Cluster analysis of the ARISA fingerprints. (b) Map of part of the Eastern Mediterranean Sea, where the stations for which ARISA was determined are delineated. Each color indicates stations belonging to the same group.

Fig 6 shows a clustering of bottom biodiversity based on DNA extraction and ARISA observed in 17 stations during MSM 13–2 cruise. A first group of microbes corresponds to populations found in relatively shallow, coastal waters (green dots in Fig 6). It is located in the Adriatic, within shelf areas. A second group (red dots) identifies abyssal biodiversities found in one of the deepest stations of the Adriatic, where Adriatic Deep Water accumulates, in two stations of the deep channel located along the Greek Ionian shelf, in three abyssal stations on the western flank of the northern Ionian basin, and in three abyssal stations of the Eastern Ionian basin: This distribution agrees well with the trajectory typically followed by Adriatic deep waters on their slow abyssal journey [4,5,7]. Finally, a third class (yellow dots) is found in the abyssal region of the central part of the northern Ionian basin, where waters of Aegean origin also accumulate [5].

## Conclusions

We envisaged the possibility of tracing routes of dense waters toward and within the ocean abyss by the use of alternative physical and biochemical parameters. In our attempt we used mercury, isotopic oxygen, biopolymeric carbon and its constituents, as well as indicators of microbial activity, and bacterial diversity found in bottom waters in the Eastern part of the Mediterranean Sea. By using these parameters, we were able to distinguish two different abyssal routes from the northern source (Adriatic Sea) and one abyssal route from the eastern source (Aegean Sea). As shown here, the two competing abyssal water masses could not be clearly discerned using the traditional approach based on temperature, salinity, and dissolved oxygen alone.

The proposed tentative approach could be especially useful in oceanic regions where competing sources of deep waters exist and/or when abyssal currents are active only episodically, to study the variability of the abyssal ocean even beyond single overflow events. In the presence of adequate abyssal bottom measurements, alternative physical, microbial, isotopic and biogeochemical parameters like those we employed in our investigation could yield valuable information also at larger spatial and temporal scales. In particular, during convective episodes, biological markers, bacteria, and microorganisms are transported to large depth and then potentially spread out into the World Ocean. Many of their physical and biological responses, however, are still largely unexplored [24]. Further aspects of the oceanic variability and underlying dynamics could be therefore inferred from their knowledge.

The explorative assessment of alternative experimental methodologies in the field of physical oceanography of the abyss seems necessary to gain information on a broader class of processes related to the formation, transformation and evolution of the water masses. The development of robust alternative experimental methodologies like the one envisaged here crucially depends on the availability of frequent and accurate data. We hope that the promising results presented here can stimulate the adoption, in future oceanographic campaigns, of an extended set of observed physical, isotopic, microbial and biogeochemical parameters similar to the one proposed here.

## Supporting Information

S1 Dataset Bottom physical and biogeochemical data collected during cruise MSM13-2. (XLS)
